# Metformin doses to ensure efficacy and safety in patients with reduced kidney function

**DOI:** 10.1371/journal.pone.0246247

**Published:** 2021-02-18

**Authors:** Isabelle H. S. Kuan, Luke C. Wilson, Jed C. Leishman, Samuel Cosgrove, Robert J. Walker, Tracey L. Putt, John B. W. Schollum, Daniel F. B. Wright

**Affiliations:** 1 School of Pharmacy, University of Otago, Dunedin, New Zealand; 2 Department of Medicine, University of Otago, Dunedin, New Zealand; University of Liège, BELGIUM

## Abstract

We aimed to develop a metformin dosing strategy to optimise efficacy and safety in patients with reduced kidney function. Metformin data from two studies stratified by kidney function were analysed. The relationship between metformin clearance and kidney function estimates was explored using a regression analysis. The maintenance dose range was predicted at different bands of kidney function to achieve an efficacy target of 1 mg/L for steady-state plasma concentrations. The dosing strategy was evaluated using simulations from a published metformin pharmacokinetic model to determine the probability of concentrations exceeding those associated with lactic acidosis risk, i.e. a steady-state average concentration of 3 mg/L and a maximum (peak) concentration of 5 mg/L. A strong relationship between metformin clearance and estimated kidney function using the Cockcroft and Gault (r^2^ = 0.699), MDRD (r^2^ = 0.717) and CKD-Epi (r^2^ = 0.735) equations was found. The probability of exceeding the safety targets for plasma metformin concentration was <5% for most doses and kidney function levels. The lower dose of 500 mg daily was required to maintain concentrations below the safety limits for patients with an eGFR of 15–29 mL/min. Our analysis suggests that a maximum daily dose of 2250, 1700, 1250, 1000, and 500 in patients with normal kidney function, CKD stage 2, 3a, 3b and 4, respectively, will provide a reasonable probability of achieving efficacy and safety. Our results support the cautious of use metformin at appropriate doses in patients with impaired kidney function.

## Introduction

Metformin is widely used in the treatment for Type 2 diabetes. It reduces blood glucose concentrations in diabetic patients while maintaining a relatively neutral effect on body weight and a low risk hypoglycaemia [1–3]. Metformin exhibits variable oral bioavailability (F), averaging about 0.55, and is largely eliminated by tubular secretion in the kidneys [4].

There is controversy regarding the safe dosing of metformin in patients with reduced kidney function. It has generally been assumed that this population will be at increased risk of metformin-associated lactic acidosis (MALA), a rare but life threatening metabolic condition with an estimated incidence of 3.3–9 cases per 100,000 patient years [5]. However, recent studies suggest that the use of metformin at appropriate therapeutic doses is unlikely to be a primary cause of lactic acidosis in many reported cases of MALA [5–7] and that suitable dose reduction in patients with impaired kidney function could mitigate the risk [5,8–10]. Unfortunately, current guidelines globally to support dosing decisions show little agreement (see a summary in S1 Table) [8,9,11–13]. The result is considerable confusion for prescribers about the safe and effective use of metformin in patients with kidney impairment.

In a recent review, we found that >50% of reported MALA cases in patients with kidney impairment were receiving daily doses that exceeded the current European Medicines Agency (EMA) recommendations by an average of 1500 mg/day [5]. This raised the possibly that reduced renal elimination of metformin may lead to metformin accumulation and an increased risk of MALA if doses are not appropriately reduced. To test this idea, we conducted simulations from a published pharmacokinetic model and found that most metformin plasma concentrations would not exceed the upper safety limit of 5 mg/L in reported MALA cases [5]. However, our simulations only examined pre-dose (trough) concentrations, the lowest values that could be measured in a patient taking metformin regularly, rather than the steady-state average concentration (C_ss,ave_) or maximum plasma concentration post-dose (C_max_). In addition, the safety limit of 5 mg/L is not well defined. Recent work by our group looking at the association between metformin plasma concentrations and severe hyperlactatemia in overdose and non-overdose patients suggests that the upper safety limit for metformin may be a C_ss,ave_ of about 3 mg/L [14], a finding broadly supported by other published work [15–18]. The upper limit of 5 mg/L has been proposed as a suitable safety metric when applied to the maximum plasma concentration after the dose (C_max_) [10,11]. Importantly, these metrics need to be clearly defined when constructing a guideline for metformin dosing in kidney impairment.

We propose that a quantitative analysis of metformin pharmacokinetics in patients with renal impairment is required to predict safe dosing based on the revised upper safety limit of 3 mg/L for C_ss,ave_ and 5 mg/L for C_max_. An important component of this is the use of the patients’ estimated kidney function to aid dose prediction. Therefore, a pragmatic guideline must also provide dosing based on different kidney function metrics that might be encountered clinically, including the commonly used creatinine-based equations; Cockcroft and Gault, Modification of Diet in Renal Disease (MDRD) and Chronic Kidney Disease Epidemiology (CKD-Epi) Collaboration [19–21].

The overarching aim of this study was to propose a dosing guideline for the safe prescribing of metformin in patients with kidney impairment (as the immediate release formulation). We conducted the analysis in three steps; (i) a quantitative analysis of metformin pharmacokinetics in patients with different levels of kidney impairment, (ii) the development of a dose banding strategy for metformin to predict dose requirements for patients with different levels of kidney impairment, and, (iii) the evaluation of the proposed dose bands using simulations from a published metformin pharmacokinetic model to predict the fraction of patients who will exceed the upper limit of safety defined as a C_ss,ave_ of 3 mg/L and/or a C_max_ of 5 mg/L.

## Materials and methods

### Pharmacokinetic analysis

Data from two studies were available for analysis, including a published pharmacokinetic analysis [13]. Full details of the study protocols and the plasma concentration assay for metformin is provided in the Supporting Information (S1 File). Ethics approval was obtained from the New Zealand health and Disability Ethics Committees (reference number MIddleMore: NTX/11/12/112 and Dunedin 14/STH/156/AM01). All patients provided written informed consent.

The pharmacokinetics of metformin were analysed by fitting the data to a published population pharmacokinetic metformin model [9] using a non-linear mixed effects methodology in NONMEM (v. 7.3). The model used was originally developed in patients with type 2 diabetes and varying degrees of kidney impairment. No formal model building or covariate analysis was conducted. Full details of the fitting procedure including an evaluation of the model fit are provided in S2 File.

The primary outputs from the pharmacokinetic analysis were individual estimates of metformin clearance. The clearance estimates were used in the next step of the analysis.

### Predicted dose bands for metformin based on kidney function metrics

Details of the methods used to determine kidney function metrics including; creatinine clearance using the Cockcroft and Gault equation (CLcr_CG_) [19], and, eGFR calculated using both the 4-variable MDRD equation (eGFR_MDRD_) [20] and the Chronic Kidney Disease Epidemiology Collaboration equation (eGFR_CKDEPI_) [21] are provided in the Supporting Information (S3 File).

The kidney dose bands were determined using a three stage analysis.

1. The relationship between metformin clearance (generated from the pharmacokinetic analysis above) and the kidney function metrics (CLcr_CG_, eGFR_MDRD_, and eGFR_CKDEPI_) was determined by linear regression in the software R (version 3.5.3). Both the eGFR_MDRD_ and eGFR_CKDEPI_ equations produce an eGFR value scaled to a body surface area (BSA) of 1.73m^2^. CLcr is not scaled to BSA. Therefore, for this analysis metformin clearance estimates were scaled to a BSA of 1.73m^2^ for comparison with eGFR_MDRD_ and eGFR_CKDEPI_ but were left unscaled for the comparison with CLcr. The predicted metformin CL (CL_predicted_ and CL_predicted_/1.73m^2^) values were determined using the regression equations given by;
  $$CL_{\mathrm{predicted}}/1.73m^{2}=b+m\times \left[eGFR_{MDRD}\;\mathrm{or}\;eGFR_{CKDEPI}\right]$$ (1)
  $$CL_{predicted}=b+m\times CLcr_{CG}$$ (2)
  Where b is the y intercept and *m* is the slope of the regression equation. Metformin CL_predicted_/1.73m^2^ and CL_predicted_ values were converted to apparent oral clearance (CL/F) values by dividing by an average bioavailability of 0.55 as reported elsewhere (4, 8).
2. The metformin CL/F_predicted_/1.73m^2^ and CL/F_predicted_ values were determined at the upper and lower bound of each Kidney Disease Improving Global Outcomes (KDIGO) kidney function band [22]. Note that CKD5 was not considered given the lack of efficacy and safety data available to support use in this population. The KDIGO kidney function bands are defined using eGFR values scaled to a BSA of 1.73m^2^. For the unscaled metformin CL/F_predicted_ values defined using CLcr we assumed the same bands, though unscaled. The kidney function bands used are summarised in Table 1.
3. The daily maintenance dose range for each kidney function band was determined from the predicted metformin CL/F_predicted_/1.73m^2^ and CL/F_predicted_ values as follows;

$$Daily\;dose\;\left(mg\right)=C_{ss,ave}\left(target\right)\times CL/F_{upper}$$ (3)

$$Daily\;dose\;\left(mg\right)=C_{ss,ave}\left(target\right)\times CL/F_{lower}$$ (4)

Where C_ss,ave_ (target) is the target steady-state average plasma concentration for metformin, CL/F_upper_ and CL/F_lower_ are the predicted CL/F_predicted_/1.73m^2^ and CL/F_predicted_ values for metformin at the upper or lower bound of the kidney function band. Note that the therapeutic range for metformin efficacy is poorly defined. We therefore chose a C_ss,ave_ (target) of 1 mg/L as a consensus (mid-point) value from several studies where values from 0.1–2 mg/L have been commonly proposed [15,16,23–25].

**Table 1:** The upper and lower values of eGFR or CLcr used to predict metformin clearance.

KDIGO kidney function band (mL/min/1.73m^2^)	eGFR_MDRD_ and eGFR_CKDEPI_ Upper and lower bound (mL/min/1.73m^2^)	CLcr_CG_ Upper and lower bound (mL/min)
>90	90–120^†^	90–120
60–89	60–89	60–89
45–59	45–59	45–59
30–44	30–44	30–44
15–29	15–29	15–29

The upper limit of the GFR range for was set as 120 mL/min.

### Evaluation of the proposed metformin dose bands

Stochastic simulations were performed to predict the range of plasma metformin concentrations expected under the proposed dose banding strategy. The simulations were conducted by implementing a published model by Duong et al [9] in R using the package RxODE (version 0.9.0–7). Details of the model and the parameter estimates used for the simulations are summarised in the Supporting Information (S2 Table in S2 File). To ensure that the model was correctly implemented we first conducted a series of trial simulations and compared these to published simulations for plasma metformin concentrations produced by Duong et al. This evaluation is summarised in S3 Fig in S4 File.

Plasma metformin concentrations were predicted over a 30 day period using the upper and lower limits of the proposed dose range for each kidney function band. One thousand virtual patients were simulated in each case. The published model included creatinine clearance normalised to 100 mL/min as a covariate to explain the between subject variability in metformin clearance rather than eGFR. The simulations were generated by sampling from a uniform distribution of CLcr values spanning each kidney function group as defined in Table 1. The kidney function values were not scaled to BSA as per Duong et al. The published model also included patient body weight as a covariate on the volume of distribution. This was fixed to 70 kilograms for the simulations (see discussion for a sensitivity analysis of different weight values). The fraction of simulated profiles on Day 30 of therapy that the exceeded safety targets for C_ss,ave_ and C_max_ of 3 mg/L and 5 mg/L respectively were determined. The dose was considered reasonable if no more than 5% of the simulated plasma concentration profiles exceeded the safety targets.

## Results

A total of 395 plasma metformin concentrations for n = 52 subjects were available for the pharmacokinetic analysis. In the regression analysis, metformin clearance values for n = 51 subjects were analysed. One subject had a highly unusual clearance value (many fold above normal) which could not be reconciled with the dosing and sampling records. This subject had little data (<2 data points above the limit of quantitation) in the terminal phase of the metformin plasma concentration profile to accurately estimate CL and so was excluded. A summary of the study subjects’ demographics are presented in Table 2.

**Table 2:** Demographics of the study subjects used in the analyses.

	Dunedin Public Hospital (n = 34)	Middlemore Hospital (n = 18)	Pooled dataset (n = 52)
Age (years)	51.5 [20.0–79.0] (32.3–66.0)	66.0 [40.0–75.0] (62.0–68.0)	61.5 [20.0–79.0] (39.3–68.0)
Sex (F:M)	5:29	3:15	8:44
Height (cm)	174 [157–195] (168–181)	172 [145–183] (168–176)	173 [145–195] (168–179)
Weight (kg)	82.1 [48.0–149.5] (75.1–87.7)	111.7 [77.2–149.8] (91.0–125.8)	85.7 [48.0–149.8] (77.6–104.4)
Body mass index (kg/m2)	26.3 [17.8–48.8] (24.2–28.9)	38.0 [25.8–51.4] (32.6–42.3)	28.9 [17.8–51.4] (25.3–35.4)
Fat-free mass (kg)	61.5 [33.9–80.5] (54.4–63.8)	68.2 [44.4–83.3] (58.8–72.8)	61.8 [33.9–83.3] (56.3–68.8)
Serum creatinine (μmol/L)	118.5 [52.0–546.0] (89.5–300.0)	259.5 [197.0–370.0] (219.3–300.0)	215.5 [52.0–546.0] (95.5–301.3)
CLcr_CG_ ^a^ (mL/min)	73.5 [9.5–167.0] (18.1–113.2)	23.4 [11.4–37.3] (19.0–28.3)	29.0 [9.5–167.0] (18.4–93.1)
eGFR_MDRD_ ^b^ (mL/min/1.73m2)	54.1 [8.9–118.5] (17.4–86.7)	20.4 [14.5–29.3] (17.7–25.7)	28.0 [8.9–118.5] (17.5–78.9)
eGFR_MDRD_ (adjusted) eGFR_MDRD_ (mL/min)	65.8 [11.0–142.7] (18.5–97.2)	24.8 [17.2–39.1] (20.8–35.8)	35.9 [11.0–142.7] (19.1–91.1)
eGFR_CKDEPI_^c^ (mL/min/1.73m^2^)	60.2 [8.2–122.4] (17.2–98.7)	20.1 [14.2–29.6] (17.3–25.8)	28.3 [8.2–122.4] (17.2–86.8)
eGFR_CKDEPI_ (adjusted) ^¶^ (mL/min)	73.2 [10.0–151.1] (17.9–109.2)	24.7 [17.0–40.4] (20.2–35.6)	35.6 [10.0–151.1] (18.6–100.8)

Data presented as median [range] (interquartile range) unless otherwise specified.

^a^ CLcr_CG_ is creatinine clearance estimated using the Cockcroft and Gault equation [19].

^b^ eGFR_MDRD_ is glomerular filtration rate estimated using the 4-variable Modification of Diet in Kidney Disease equation [20].

^c^ eGFR_CKDEPI_ is glomerular filtration rate estimated using the Chronic Kidney Disease Epidemiology Collaboration equation [21].

^¶^Note that eGFR_MDRD_ (adjusted) and eGFR_CKDEPI_ (adjusted) were adjusted for the individual body surface area measurements for each subject calculated using the Du Bois Method.

### Predicted dose bands for metformin

The regression analysis of metformin clearance and each kidney function metric (CLcr_CG_, eGFR_MDRD_, and eGFR_CKDEPI_) is presented in S4 Fig and S4 Table in S5 File.

The final dose prediction equations for metformin using CLcr_CG_, eGFR_MDRD_, and eGFR_CKDEPI_ are given as follows;

$$Daily\;dose\;\left(mg\right)=C_{ss,ave\left(target\right)}\cdot \left(6.81+6.34\cdot CLcr_{CG}\right)\cdot 24$$ (5)

$$Daily\;dose\;\left(mg\right)=C_{ss,ave\left(target\right)}\cdot \left(3.99+7.21\cdot eGFR_{MDRD}\right)\cdot 24$$ (6)

$$Daily\;dose\;\left(mg\right)=C_{ss,ave\left(target\right)}\cdot \left(4.76+6.45\cdot eGFR_{CKDEPI}\right)\cdot 24$$ (7)

Note that CLcr_CG_, eGFR_MDRD_, and eGFR_CKDEPI_ are expressed in L/h or L/h/1.73m^2^ in these equations.

The predicted dose range for each kidney function band at the upper and lower bounds are presented in Table 3. Doses were rounded assuming the availability of immediate release tablets of 500 mg and 850 mg. For the purposes of this analysis, it was assumed that the 500 mg tablet could be split to produce a 250 mg dose.

**Table 3:** Predicted metformin maintenance doses at the upper and lower bounds of each CKD category.

Kidney function bands (mL/min/1.73m^2^ or mL/min)	Predicted daily metformin dose range (mg)		
	Using CLcr_CG_^a^	Using eGFR_MDRD_^b^	Using eGFR_CKDEPI_^c^
90–120	1700–2250	1700–2250	1700–2250
60–89	1250–1700	1250–1700	1250–1700
45–59	1000–1250	1000–1250	1000–1250
30–44	750–1000	750–1000	750–1000
15–29	500–750	500–750	500–750

^a^ using Eq 5

^b^ using Eq 6

^c^ using Eq 7.

### Evaluation of the proposed metformin dose bands

**Fig 1:**
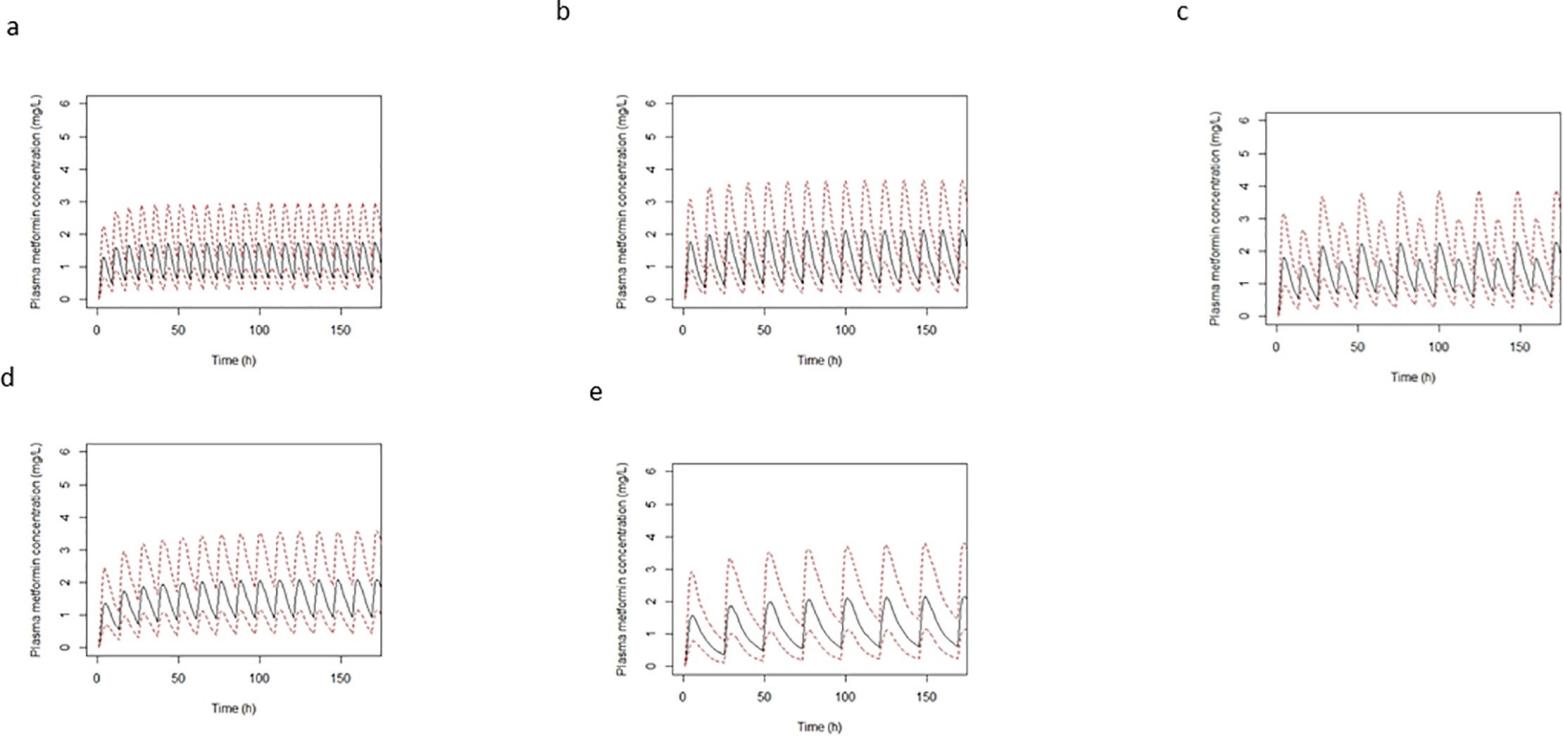
Simulated plasma metformin concentration versus time profiles for select doses in each kidney function band (immediate release formulation); (a) 750 mg TID normal kidney function, (b) 850 mg BID CKD2, (c) 750 mg mane, 500 mg nocte, CKD3a, (d) 500 mg BD, CKD3b, (e) 500 mg OD CKD4.

The fraction of model-predicted metformin concentrations exceeding the C_ss,ave_ and C_max_ targets are shown in Table 4. The doses resulted in metformin plasma concentrations that exceeded C_ss,ave_ of 3 mg/L or a C_max_ of 5 mg/L less 5% of the time with the exception of the upper dose range proposed for the kidney function band 15–29 mL/min (i.e. 500mg daily). The simulated plasma metformin concentration profiles for each predicted daily dose is presented in Fig 1.

**Table 4:** The fraction of predicted metformin C_ss,ave_ and C_max_ concentrations exceeding the safety targets.

Kidney function band (mL/min/1.73m^2^ or mL/min)	Daily dose (mg)	Simulated dose (mg)	Fraction of C_ss,ave_ >3 mg/L	Fraction of C_max_ >5 mg/L
90–120	2250	750 TID	0.006	0.001
	1700	850 BD	0.001	0.001
60–89	1700	850 BD	0.015	0.009
	1250	750 mane, 500 nocte	0.003	0.001
45–59	1250	750 mane, 500 nocte	0.014	0.012
	1000	500 BD	0.003	0.000
30–44	1000	500 BD	0.031	0.008
	750	750 OD	0.006	0.021
15–29	750	750 OD	0.149	0.122
	500	500 OD	0.020	0.014

OD: Once a day. BD: Twice a day, TID: Three times a day, *mane*: *Morning*, *nocte*: *Evening*.

## Discussion

The principal output from the study is a dose banding method based on kidney function for metformin doses. The under pinning assumption is that dose reduction in patients with poor kidney function to maintain plasma concentrations below those associated with severe hyperlactatemia should mitigate the risk of lactic acidosis. By extension, the expected glucose lowering effect for the doses proposed should be normalised across the CKD bands. Our dose bands suggest a maximum metformin daily dose of 2250, 1700, 1250, 1000, and 500 in patients with normal kidney function, CKD stage 2, 3a, 3b and 4 respectively. The upper dose limit predicted for both the CKD stage 4 band (750mg daily) was found to produce concentrations above the C_ss,ave_ and C_max_ safety limits in 12–15% and 20–44% of patients. Therefore the lower dose of 500mg once daily would be required to maintain concentrations within the safety margins for patients with CKD 4 according to our results. Further, given the observed variability between people in metformin pharmacokinetics [9], the maximum doses proposed here may need to be supported by plasma metformin measurements to assist dose individualisation. It is important to note that our dose predictions are intended for stable CKD. We do not advocate the use of metformin in patients with unstable kidney impairment, those at high risk of acute kidney injury, or inpatients with CKD5 where there is currently limited data to support safety and efficacy.

Several previous studies have explored the dosing of metformin in patients with poor kidney function. In a dose-escalation study, Dissanayake et al administered increasing doses of 250mg, 500mg and 1000mg daily to patients with CKD 4 [13]. Median metformin plasma concentrations were 0.08, 0.239 and 1.9 mg/L, and median *C_max_* values were reported to be 0.76, 1.13, and 2.28 mg/L for 250, 500mg, and 1000mg daily respectively. Similarly, Lalau et al performed a metformin dose-finding study in patients with CKD stage 3–5 using doses of 500 mg– 2000 mg daily [10]. At the highest dose level, about 25% of the C_ss,ave_ values were above the upper limit of safety. We note that this dose level would not be recommended using our dose banding guidelines unless the patient had a reported eGFR > 60mL/min/1.73m^2^. In addition, Lalau et al used a slightly more conservative C_ss,ave_ target of 2.5mg/L. It is noteworthy that the revised dosing guideline from the EMA [12] includes a dose of 2000 mg daily in patients with CKD stage 3a (45–59 mL/min/1.73m^2^), a dose that would exceeds the recommendation in our guideline (i.e. 1250mg daily).

Both Dissanayake et al and Lalau et al used observed metformin concentrations in prospective studies to draw conclusions about the safe doses to use in patients with poor kidney function [10,13]. By contrast, Duong et al developed a population PK model for metformin based on data from n = 304 patients and used the model to predict plasma concentrations across different level of kidney function using Monte Carlo simulation [9]. The authors determined that the probability of concentrations exceeding the C_max_ target of 5mg/L were minimal using a maximum dose 500mg, 1000mg, 2000mg, and 3000mg daily for patients with CLcr of 15, 30, 60, 120 mL/min, respectively. These dose recommendations align roughly with those predicted by our dose-bands, although our scheme offers a range of doses across the CKD groups.

One strength of our study is the use of metformin C_ss,ave_ efficacy and safety targets as a basis for the dosing guideline. While the lower end of the therapeutic range for metformin is not well defined, it is generally agreed that steady state concentrations between 0.1–2 mg/L are needed to achieve adequate blood glucose lowering [15,16,23–25]. By targeting a mid-point in this range, our dose guideline should optimise efficacy while maintaining safety. The simulations from the published model suggest that this is accomplished while maintaining concentrations below those associated with hyperlactatemia. Indeed, the use of a robust Monte Carlo simulation methodology and a published population PK model to demonstrate the utility of our dose bands is another strength of this work.

The results of this analysis should be viewed in light of some limitations. While dose dependant absorption and variable oral bioavailability may occur with metformin at higher doses [26,27], we are not able to determine if this might impact the dose recommendations proposed here. We also based our dose predictions on a univariate analysis of the relationship between kidney function and metformin clearance. Other potential covariates such as body size and kidney transporter genotypes were not considered. To address this limitation, a sensitivity analysis was conducted to test the influence of weight on the dosing recommendations. Simulations from the population PK model at the extremes of weight observed in the population analysed by Duong et al (41kg and 165kg) had only a negligible impact on the predicted average steady-state plasma concentrations suggesting that the primary covariate that determined metformin dose requirements is kidney function. It is noteworthy that body weight has not been consistently found to influence dosing requirements in other published work [8]. In addition, we assumed a fixed bioavailability of 0.55 for this work based on a published value. Bioavailability may be altered in CKD patients and indeed may have contributed to the higher than expected plasma concentrations for CKD stage 4 patients in the simulations conducted. Finally, we base the *C_ss,ave_* target for safety on a retrospective analysis looking at the observed association between metformin concentrations and serum lactate [14], not on the occurrence of lactic acidosis. We acknowledge that toxicology data suggests a much higher threshold for metformin concentrations and lactic acidosis, e.g. 10mg/L [28]. While the cause and effect relationship between metformin plasma concentrations, renal impairment, serum lactate, and lactic acidosis is not well understood, we chose our safety target to be conservative and assumed that severe hyperlactatemia acts as a surrogate for lactic acidosis risk. The use of serum lactate in this way does not allow us to distinguish between metformin-induced, metformin-associated, or metformin-unrelated lactic acidosis, as proposed by Lalau et al [29]. In addition, it is not possible to determine from the research conducted here if lactate itself could be used to guide metformin dosing.

A dosing guideline for metformin based on kidney function was developed and evaluated. Our dose bands suggest a maximum metformin daily dose of 2250, 1700, 1250, 1000, and 500 in patients with normal kidney function, CKD stage 2, 3a, 3b and 4, respectively. Predictions from a published PK model for metformin under our dose bands suggest that the proposed upper limit of safety for C_ss,ave_ and C_max_ targets will not be exceeded in the majority of patients. Our results support the cautious of use metformin at appropriate doses in patients with impaired kidney function.

## Supporting information

S1 Table Summary of published renal dosing guidelines and contraindications for metformin. (DOCX)

S1 File Study details for the analysed data. (DOCX)

S2 File Pharmacokinetic analysis of the metformin data. S1 Fig. Schematic of the published Duong et al model. S2 Fig. Model fit (pcVPCs) for the Dunedin data (top) and Middlemore data (bottom). S2 Table. Parameter values for the final published metformin model by Duong et al. S3 Table. Parameter values estimated from the Dunedin and Middlemore data.
(DOCX)

S3 File Determination of creatinine clearance and eGFR metrics. (DOCX)

S4 File Evaluation of the implemented PK model. S3 Fig. Predicted plasma metformin concentrations at varying levels of renal impairment.
(DOCX)

S5 File Regression analysis details. S4 Fig. The clearance for metformin regressed against different measures of kidney function, including (a) CLcrCG, (b) eGFR_MDRD_, and, (c) eGFR_CKDEPI._ S4 Table. Summary of linear regression results.
(DOCX)

## References

[1] Golay A. Metformin and body weight. Int J Obes. 2008;32:61–72. 10.1038/sj.ijo.0803695 17653063

[2] Campbell IW, Howlett HC. Worldwide experience of metformin as an effective glucose-lowering agent: a meta-analysis. Diabetes Metab Revi. 1995;11 (Supp 1):S57–62. 10.1002/dmr.5610110509 8529486

[3] UK Prospective Diabetes Study (UKPDS) Group. Effect of intensive blood-glucose control with metformin on complications in overweight patients with type 2 diabetes (UKPDS 34). Lancet. 1998;352(9131):854–65. 9742977

[4] Graham GG, Punt J, Arora M, Day RO, Doogue MP, Duong JK et al Clinical pharmacokinetics of metformin. Clin Pharmacokinet. 2011;50:81–98. 10.2165/11534750-000000000-00000 21241070

[5] Kuan IHS, Savage RL, Duffull SB, Walker RJ, Wright DFB. The association between metformin therapy and lactic acidosis. Drug Saf. 2019;42:1449–69. 10.1007/s40264-019-00854-x 31372935

[6] Stades AME, Heikens JT, Erkelens DW, Holleman F, Hoekstra JBL. Metformin and lactic acidosis: Cause or coincidence? A review of case reports. J Intern Med. 2004;255:179–87. 10.1046/j.1365-2796.2003.01271.x 14746555

[7] Salpeter SR, Greyber E, Pasternak GA, Salpeter EE. Risk of fatal and nonfatal lactic acidosis with metformin use in type 2 diabetes mellitus. Cochrane Database Syst Rev. 2010(1):CD002967 10.1002/14651858.CD002967.pub3 20091535

[8] Duong JK, Kroonen M, Kumar SS, Heerspink HL, Kirkpatrick CM, Graham GG, et al A dosing algorithm for metformin based on the relationships between exposure and renal clearance of metformin in patients with varying degrees of kidney function. Eur J Clin Pharmacol. 2017;73:981–90. 10.1007/s00228-017-2251-1 28451709

[9] Duong JK, Kumar SS, Kirkpatrick CM, Greenup LC, Arora M, Lee TC, et al Population pharmacokinetics of metformin in healthy subjects and patients with type 2 diabetes mellitus: simulation of doses according to renal function. Clin Pharmacokinet. 2013;52:373–84. 10.1007/s40262-013-0046-9 23475568

[10] Lalau JD, Kajbaf F, Bennis Y, Hurtel-Lemaire AS, Belpaire F, De Broe ME. Metformin Treatment in Patients With Type 2 Diabetes and Chronic Kidney Disease Stages 3A, 3B, or 4. Diabetes Care. 2018;41:547–53. 10.2337/dc17-2231 29305402

[11] Briston-Meyers Squibb Company. GLUCOPHAGE (metformin hydrochloride) tablets. Princeton, USA. 2017. https://packageinserts.bms.com/pi/pi_glucophage_xr.pdf. Accessed 10 August 2018.

[12] European Medicines Agency. Use of metformin to treat diabetes now expanded to patients with moderately reduced kidney function. 2016. https://www.ema.europa.eu/en/news/use-metformin-treat-diabetes-now-expanded-patients-moderately-reduced-kidney-function. Accessed 1 Jan 2021.

[13] Dissanayake AM, Wheldon MC, Ahmed J, Hood CJ. Extending Metformin Use in Diabetic Kidney Disease: A Pharmacokinetic Study in Stage 4 Diabetic Nephropathy. Kidney Int Rep. 2017;2:705–12. 10.1016/j.ekir.2017.03.005 29318219 PMC5720630

[14] Kuan IHS, Wright DFB, Duffull SB, Zhu X. Understanding the association between metformin plasma concentrations and lactate. Br J Clin Pharmacol. 2020; 10.1111/bcp.14394 32519376

[15] Kajbaf F, De Broe ME, Lalau JD. Therapeutic Concentrations of Metformin: A Systematic Review. Clin Pharmacokinet. 2016;55:439–59. 10.1007/s40262-015-0323-x 26330026

[16] Lalau JD, Lacroix C. Measurement of metformin concentration in erythrocytes: clinical implications. Diabetes Obes Metab. 2003;5:93–8. 10.1046/j.1463-1326.2003.00241.x 12630933

[17] Boucaud-Maitre D, Ropers J, Porokhov B, Altman JJ, Bouhanick B, Doucet J, et al Lactic acidosis: relationship between metformin levels, lactate concentration and mortality. Diabet Med. 2016;33:1536–43. 10.1111/dme.13098 26882092

[18] Smith FC, Stocker SL, Danta M, Carland JE, Kumar SS, Liu Z, et al The safety and pharmacokinetics of metformin in patients with chronic liver disease. Aliment Pharmacol Ther. 2020;51:565–75. 10.1111/apt.15635 31960986

[19] Cockcroft DW, Gault MH. Prediction of creatinine clearance from serum creatinine. Nephron. 1976;16:31–41. 10.1159/000180580 1244564

[20] Levey AS, Coresh J, Greene T, Stevens LA, Zhang YL, Hendriksen S, et al Using standardized serum creatinine values in the modification of diet in renal disease study equation for estimating glomerular filtration rate. Ann Intern Med. 2006;145:247–54. 10.7326/0003-4819-145-4-200608150-00004 16908915

[21] Levey AS, Stevens LA, Schmid CH, Zhang YL, Castro AF, Feldman HI, et al A new equation to estimate glomerular filtration rate. Ann Intern Med. 2009;150:604–12. 10.7326/0003-4819-150-9-200905050-00006 19414839 PMC2763564

[22] KDIGO 2012 Clinical Practice Guideline for the Evaluation and Management of Chronic Kidney Disease. Kidney Int (Supplements). 2013;3:1–150. 10.1038/ki.2013.243 23989362

[23] Lalau JD, Lacroix C, Compagnon P, De Cagny B, Rigaud JP, Bleichner G, et al Role of metformin accumulation in metformin-associated lactic acidosis. Diabetes Care. 1995;18:779–84. 10.2337/diacare.18.6.779 7555503

[24] Scheen AJ. Clinical pharmacokinetics of metformin. Clin Pharmacokinet. 1996;30:359–71. 10.2165/00003088-199630050-00003 8743335

[25] Lalau JD, Lemaire-Hurtel AS, Lacroix C. Establishment of a database of metformin plasma concentrations and erythrocyte levels in normal and emergency situations. Clin Drug Investig. 2011;31:435–8. 10.2165/11588310-000000000-00000 21401215

[26] Tucker GT, Casey C, Phillips PJ, Connor H, Ward JD, Woods HF. Metformin kinetics in healthy subjects and in patients with diabetes mellitus. Br J Clin Pharmacol. 1981;12:235–46. 10.1111/j.1365-2125.1981.tb01206.x 7306436 PMC1401849

[27] Sambol NC, Brookes LG, Chiang J, Goodman AM, Lin ET, Liu CY, et al Food intake and dosage level, but not tablet vs solution dosage form, affect the absorption of metformin HCl in man. Br J Clin Pharmacol. 1996;42:510–2. 10.1111/j.1365-2125.1996.tb00017.x 8904626

[28] Bennis Y, Bodeau S, Batteux B, Gras-Champel V, Masmoudi K, Maizel J, et al A Study of Associations Between Plasma Metformin Concentration, Lactic Acidosis, and Mortality in an Emergency Hospitalization Context. Crit Care Med. 2020 10.1097/ccm.0000000000004589 33003077

[29] Lalau JD, Kajbaf F, Protti A, Christensen MM, De Broe ME, Wiernsperger N. Metformin-associated lactic acidosis (MALA): Moving towards a new paradigm. Diabetes Obes Metab 2017;19:1502–12. 10.1111/dom.12974 28417525

