# Supplementary material for: Metformin doses to ensure efficacy and safety in patients with reduced kidney function
Source: PLoS One. 2021 Feb 18;16(2):e0246247. doi: 10.1371/journal.pone.0246247 (PMC7891741; doi:10.1371/journal.pone.0246247)
Supplement: S1 Table — (DOCX) [file pone.0246247.s001.docx]

S1 Table. Summary of metformin renal dosing guidelines and contraindications

| Country | Ref | Year | Renal estimation method | Renal dose adjustment |  | Renal contraindication |
| --- | --- | --- | --- | --- | --- | --- |
|  |  |  |  | Renal function | Recommended dose |  |
| Australia | [1] | 2016 | CLcr |  |  | <60 mL/min |
|  | [2] | 2016 | eGFR | 30-60 mL/min/1.73m^2^ | Use with caution  Reduce dose | <30 mL/min/1.73m^2^ |
|  | [3] | 2016 | CLcr | 60-90 mL/min  30-60 mL/min | Maximum: 2000 mg/day  Maximum: 1000 mg/day | <30 mL/min |
|  | [4] | 2017 | CLcr | <90 mL/min  15-30 mL/min | Reduce maximum dose  Metformin may be considered for patients with stable renal function | 15 mL/min |
| Canada | [5] | 2015 | eGFR | <60 mL/min | Reduce dose | - |
|  | [6] | 2015 | eGFR | 30-59 mL/min  ≥60 mL/min | Reduce dose  No dose adjustment | ≤29 mL/min |
|  | [7] | 2016 | CLcr  eGFR | <60 mL/min | Caution | <30 mL/min |
| New Zealand | [8] | 2014 | CLcr | 60-120 mL/min  30-60 mL/min  15-30 mL/min | Maximum: 2000 mg/day  Maximum: 1000 mg/day  Maximum: 500 mg/day | <15 mL/min |
|  | [9] | 2015 | CLcr | 60-120 mL/min  30-60 mL/min  15-30 mL/min | Maximum: 2000 mg/day  Maximum: 1000 mg/day  Maximum: 500 mg/day | <15 mL/min |
|  | [10] | 2015 | - | 60-120 mL/min  30-60 mL/min  15-30 mL/min | Maximum: 2000 mg/day  Maximum: 1000 mg/day  Maximum: 500 mg/day | <15 mL/min |
|  | [11] | 2015 | eGFR | >90 mL/min/1.73m^2^  60-90 mL/min/1.73^2^  30-60 mL/min/1.73^2^  <30 mL/min/1.73^2^ | Maximum: 3000 mg/day  Maximum: 2000 mg/day  Maximum: 1000 mg/day  Discuss with specialist | Quantitative – *significant impairment or renal failure* |
|  | [12] | 2016 | CLcr | 60-120 mL/min  30-60 mL/min  15-30 mL/min | Maximum: 2000 mg/day  Maximum: 1000 mg/day  Maximum: 500 mg/day | <15 mL/min |
|  | [13] | 2017 | eGFR | 60-120 mL/min/1.73m^2^  30-60 mL/min/1.73^2^  15-30 mL/min/1.73^2^ | Maximum: 2000 mg/day  Maximum: 1000 mg/day  Maximum: 500 mg/day | <15 mL/min/1.73m^2^ |
|  | [14] | 2020 | CLcr | 60-120 mL/min  30-60 mL/min  15-30 mL/min | Maximum: 2000 mg/day  Maximum: 1000 mg/day  Maximum: 500 mg/day | <15 mL/min |
| United States of America | [15] | 2016 | eGFR | 30-45 mL/min/1/73m^2^ | Not recommended  Reassess benefit to risk | 30 mL/min/1.73m^2^ |
|  | [16] |  |  |  | Dose individualisation | Serum creatinine above normal upper limit for age  Males: ≥1.5 mg/dL  Females: ≥1.4 mg/dL |
|  | [17] | 2016 | eGFR | 30-45 mL/min/1.73m^2^ | Dose reduction | Stop medication if GFR is low |
|  | [18] | 2017 | eGFR | 45-60 mL/min/1.73m^2^  30-44 mL/min/1.73m^2^  <30 mL/min/1.73m^2^ | Consider dose adjustments  Consider dose adjustments  Referral to a nephrologist | <30 mL/min/1.73m^2^ |
|  | [19] | 2020 | eGFR | <45 mL/min/1.73m^2^ | Reassess metformin use | <30 mL/min/1.73m^2^ |
| United Kingdom | [20] | 2015 | eGFR | 45 mL/min/1.73m^2^ | Review dose | 30 mL/min/1.73m^2^ |
|  | [21] | 2015 | CLcr  eGFR | 45-59 mL/min  45-59 mL/min/1.73m^2^ | Initial dose: 500 or 850 mg OD  Maximum: 2000 mg/day | - |
|  | [22] | 2016 | GFR | 60-89 mL/min  45-59 mL/min  30-44 mL/min | Maximum: 3000 mg/day  Maximum: 2000 mg/day  Maximum: 1000 mg/day | <30 mL/min |

CLcr stands for creatinine clearance, GFR stands for glomerular filtration rate, eGFR stands for estimated glomerular filtration rate. Note that the renal estimation method recommended to guide the renal dosing of metformin were copied verbatim from the dosing guidelines and do not elicitly state how CLcr, GFR or eGFR were measured/calculated.

**References**

1. Alphapharm. Product information - Diabex XR - Metformin Hydrochloride. 2016. <https://gp2u.com.au/static/pdf/D/DIABEX_XR-PI.pdf>. Accessed 10 May 2017.

2. Diabetes Australia. General practice management of type 2 diabetes. 2016. <https://static.diabetesaustralia.com.au/s/fileassets/diabetes-australia/5d3298b2-abf3-487e-9d5e-0558566fc242.pdf>. Accessed 11 April 2017.

3. Australian Medicines Handbook Pty Ltd. Australian Medicines Handbook 2016. Rundle Mall, SA, Australia: Australian Medicines Handbook Pty Ltd; 2016.

4. Australian Medicines Handbook Pty Ltd. Australian Medicines Handbook 2017. Rundle Mall, SA, Australia: Australian Medicines Handbook Pty Ltd; 2017.

5. British Columbia Guidelines. Diabetes Care. 2015. <https://www2.gov.bc.ca/assets/gov/health/practitioner-pro/bc-guidelines/diabetes_care_full_guideline.pdf>. Accessed 7 April 2017.

6. Canadian Diabetes Association. Canadian Diabetes Association Guidelines - appendix 6. 2015. <http://guidelines.diabetes.ca/browse/appendices/appendix6_2015>. Accessed 10 April 2017.

7. Canadian Diabetes Association. Canadian Diabetes Association Guidelines 2016. 2016. <http://guidelines.diabetes.ca/cdacpg_resources/Ch13_Table1_Antihyperglycemic_agents_type_2_nov-2016.pdf>. Accessed 10 April 2017.

8. MYLAN. METFORMIN MYLAN. New Zealand. 2014. <https://www.medsafe.govt.nz/profs/datasheet/m/MetforminMylantabs.pdf>. Accessed 11 April 2017.

9. Metchek New Zealand Data Sheet. New Zealand. 2015. <https://www.medsafe.govt.nz/profs/datasheet/m/metchektab.pdf>. Accessed 10 May 2017.

10. Douglas Pharmacuticals Limited. Metformin Generic Health. New Zealand. 2015. <http://www.medsafe.govt.nz/profs/datasheet/m/MetforminGenericHealthtab.pdf>. Accessed 10 May 2017.

11. SAFERX. Metformin - Safe prescribing - First-line. New Zealand. 2015. <http://www.saferx.co.nz/full/Metformin.pdf>. Accessed 10 April 2017.

12. APOTEX NZ LTD. Metformin. 2016. <http://www.medsafe.govt.nz/profs/Datasheet/m/Metformintab.pdf>. Accessed 7 April 2017.

13. New Zealand Formulary. Metformin Hydrochloride. 2017. <http://nzf.org.nz/nzf_3715>. Accessed 10 April 2017.

14. New Zealand Formulary. Metformin hydrochloride. 2020. <https://nzf.org.nz/nzf_3715>. Accessed 22 November 2020.

15. US Food and Drug Administration. FDA Drug Safety Communication: FDA revises warnings regarding use of the diabetes medicine metformin in certain patients with reduced kidney function. 2016. <https://www.fda.gov/Drugs/DrugSafety/ucm493244.htm>. Accessed 27/06 2018.

16. Briston-Meyers Squibb Company. GLUCOPHAGE (metformin hydrochloride) tablets. Princeton, USA. 2017. <https://packageinserts.bms.com/pi/pi_glucophage_xr.pdf>. Accessed 10 August 2018.

17. American Diabetes Association. Standards of Medical Care in Diabetes - 2016. The Journal of Clinical and Applied Research and Education. 2016;39 (Supp 1).

18. American Diabetes Association. Standards of Medical Care in Diabetes - 2017. The Journal of Clinical and Applied Research and Education. 2017;40 (Supp 1).

19. American Diabetes Association. Standards of Medical Care in Diabetes - 2020 Abridged for Primary Care Providers. Clinical Diabetes. 2020;38(1):10-38.

20. National Institute for Health and Care Excellence. Type 2 diabetes in adults: management 2015. 2015. <https://www.nice.org.uk/guidance/ng28/resources/type-2-diabetes-in-adults-management-pdf-1837338615493>. Accessed 10 April 2017.

21. MIMS UK. Metformin use in renal impairment extended 2015. 2015. <https://www.medicines.org.uk/emc/medicine/1043>. Accessed 10 April 2017.

22. Glucophage 500 mg and 850 mg film coated tablets. 2016. <https://www.medicines.org.uk/emc/medicine/1043>. Accessed 10 April 2017.
