# Supplementary material for: Metformin doses to ensure efficacy and safety in patients with reduced kidney function
Source: PLoS One. 2021 Feb 18;16(2):e0246247. doi: 10.1371/journal.pone.0246247 (PMC7891741; doi:10.1371/journal.pone.0246247)
Supplement: S1 File — (DOCX) [file pone.0246247.s002.docx]

**S2 File. Study details for the analysed data**

**Dunedin Public Hospital metformin study**

This was an observational study conducted by the Nephrology research unit, Department of Medicine, University of Otago, New Zealand. The study was registered with the Australian and New Zealand Clinical Trials Registry (ANZCTR), number: ACTRN12614001180616. All study subjects gave written and informed consent.

Participants were metformin-naïve individuals > 18 years of age who were not currently receiving any form of renal replacement therapy. The study was stratified to recruit participants across a range of different renal function levels defined by three groups; 1) a baseline estimated glomerular filtration rate (eGFR) of ≥ 60mL/min, 2) baseline eGFR of ≥ 30mL/min and < 60mL/min, and, 3) baseline eGFR of < 30mL/min. Individuals were excluded if they; were unable or unwilling to give written informed consent, had a diagnosis of Type 2 diabetes, were taking metformin prior to the study, had a >25% change in eGFR in the past month, were pregnancy, had a known allergy to medications used in the study (i.e. biguanides or aminoglycosides), were clinically instable (e.g. fragile fluid balance) or, were taking drugs known or a risk for interacting with the renal tubular transport of metformin or creatinine (e.g. some beta-lactam antibiotics, atenolol, calcium channel blockers, antiarrhythmic drugs, histamine (H_2_) antagonists, thiazide diuretics, antituberculosis drugs and probenecid). During the eligibility screening process baseline information, including study participant demographics and concomitant medications were recorded.

Study participants fasted overnight prior to the first study day. On the first study day, a blood sample was taken for the measurement of baseline plasma metformin and creatinine. Subjects then received a single dose of metformin 500 mg orally with 250 mL of filtered water. Blood samples were collected at the following times post drug administration: 15-30 minutes, 30-60 minutes, 90-120 minutes, 3 hours, 5-6 hours, 8-12 hours and 24 hours for the measurement of metformin and creatinine. An additional blood sample was collected at 30-36 hours in some individuals for the measurement of metformin. Timed urine samples were collected at times 0-3 hours, 3-8 hours and 8-24 hours post drug administration. The collected blood and urine samples were stored at -80^o^C.

**Middlemore Hospital metformin study**

Data from an open-label, prospective, phase I, safety study conducted at Middlemore Hospital (Auckland, New Zealand) was available for analysis and is described in detail elsewhere [1]. The study was approved by the New Zealand Health and Disability Ethics Committees, reference number: NTX/11/12/112. All patients provided written and informed consent.

Eighteen patients with type 2 diabetes mellitus and stable stage 4 chronic kidney disease (CKD) were enrolled in the study. Subjects were included in the study if they were: 30-75 years of age, had a diagnosis of type two diabetes for at least two years, an HbA1c level between 6% and 11%, and, stable stage 4 CKD. Stage 4 CKD was defined as a stable eGFR value between 15-30 mL/min/1.73m^2^ over the preceding three months. Subjects were excluded if they presented with: a history of metformin intolerance, pregnancy, breastfeeding, pre-existing metabolic acidosis or having significant risk factors for metabolic acidosis. Significant risk factors for metabolic acidosis included: morbid obesity (>160 kg), unstable ischemic heart disease, a planned radiocontrast examination within the following six months and/or relevant medical comorbidities (e.g. severe chronic obstructive pulmonary disease, unstable congestive heart failure and significant liver disease).

Study participants were randomised into one of three study arms to receive either 250, 500 or 1000 mg of metformin orally once daily for four weeks. Participants randomised to receive the lowest dose of metformin were the first to complete the study. Participants in the second and third study arm then completed the study if metformin safety and tolerability were reported to be satisfactory, respectively.

Study participants fasted overnight prior to the first study day. On the first study day, blood samples were collected to assess baseline fasting metabolic control profiles (glucose, insulin, lipids and HbA1c) and a safety profile (serum lactate, bicarbonate, venous pH, renal function, electrolytes, liver enzymes and a full blood count). A continuous capillary glucose monitoring system was used to assess glycaemic control over a 72 hour period. On study day four, study participants received a single daily dose of metformin followed by a standard breakfast. Blood samples were taken at 0 (baseline), 2, 4, 6, 8 and 24 hours after the first dose of metformin was administrated. Study participants continued their daily metformin therapy for four weeks, returning to the clinic on a weekly basis to measure trough metformin concentrations, monitor their safety profiles and to assess for adverse events. The 72 hour continuous capillary glucose monitoring system was repeated on the last three days of the study and HbA1c was measured on the last day of the study.

**Metformin plasma concentration assay**

Metformin plasma concentrations were measured using a high-performance liquid chromatography (HPLC) assay described and validated by Zhang et al for both studies (21). Concentrations were found to be linear over the concentration range of 0.02-4 mg/L while the lower limit of quantification was reported to be 0.02 mg/L. The intra- and inter-day coefficients of variation were reported to be less than 9.0%.

**Creatinine assay**

Serum creatinine was measured as part of routine blood work by Southern Community Labs (Dunedin Study) and Counties Manukau Health lab services (Middlemore study) using an assay traceable to a reference method based on isotope dilution-mass spectrometry (IDMS).

**References**

1. Dissanayake AM, Wheldon MC, Ahmed J, Hood CJ. Extending Metformin Use in Diabetic Kidney Disease: A Pharmacokinetic Study in Stage 4 Diabetic Nephropathy. Kidney Int Rep 2017;2(4):705-12. doi:10.1016/j.ekir.2017.03.005.
