# Supplementary material for: Metformin doses to ensure efficacy and safety in patients with reduced kidney function
Source: PLoS One. 2021 Feb 18;16(2):e0246247. doi: 10.1371/journal.pone.0246247 (PMC7891741; doi:10.1371/journal.pone.0246247)
Supplement: S2 File — S1 Fig. Schematic of the published Duong et al model. S2 Fig. Model fit (pcVPCs) for the Dunedin data (top) and Middlemore data (bottom). S2 Table. Parameter values for the final published metformin model by Duong et al. S3 Table. Parameter values estimated from the Dunedin and Middlemore data. (DOCX) [file pone.0246247.s003.docx]

**S2 File. Pharmacokinetic analysis of the metformin data**

The pharmacokinetics of metformin were assessed by fitting the data to a published population pharmacokinetic metformin model [1] using a non-linear mixed effects methodology in NONMEM (v. 7.3). The first-order conditional and interaction estimation method was used. The convergence criterion was set to three significant digits. An Intel-5 processor and a GNU Fortran 95 compiler (GCC 4.6.0) were employed for the analysis. The model runs were executed using PsN v 4.8.0. Pre- and post-processing was conducted using R v3.5.3 (The R Foundation).

The published model was originally developed in Type 2 diabetic patients with varying degrees of renal impairment.

**Description of published metformin model**

The pharmacokinetics of metformin were described by a two-compartment model with first-order absorption for immediate release formulations (Figure S1). In the model, creatinine clearance and total body weight were found to be significant covariates on the apparent clearance (CL/F) and central volume compartment (V_1_/F) for metformin, respectively. The published equations for CL/F and V_1_/F are as follows:

${CL}/F=\left( \theta_{CL} \times\left( {{CL}_{CR}}/6 \right) \right)\times e^{PPVCL}$ (S1)

${V_{1}}/F=\left( \theta_{V_{1}} \times\left( {TBW}/{70} \right) \right)\times e^{PPVV1}$ (S2)

where Θ_CL_ is the mean population value for CL/F, Θ_V1_ is the mean population value for V_1_/F, PPVCL is the inter-individual variability and inter-occasion variability for CL/F, PPVV1 is the inter-individual variability inter-individual variability for V_1_/F, CLcr is creatinine clearance, and, TBW is total body weight.

S1 Figure. Schematic of the published Duong et al model

S2 Table. Parameter values for the final published metformin model by Duong et al

| Parameter | Values |
| --- | --- |
| CL/F (L/h) | 72 |
| V_1_/F (L) | 149 |
| Q/F (L/h) | 12.18 |
| V_2_/F (L) | 182 |
| k_a_ (h^-1^) | 0.35 |
| TLAG (h) | 0.38 |
| Inter-individual variability | |
| ηCL (CV%) | 34.4 |
| ηV_1_ (CV%) | 54.1 |
| ηQ (CV%) | 66.0 |
| ηV_2_ (CV%) | 75.4 |
| Covariance of parameters (R) | |
| ηCL, ηV_1_ | 0.505 |
| ηCL, ηQ | 0.710 |
| ηCL, ηV_2_ | 0.688 |
| Residual error | |
| σ_add_ (mg/L) | 0.018 |
| σ_prop_ (%) | 0.271 |

CL/F apparent clearance, V_1_/F apparent central volume compartment, Q/F apparent inter-compartmental clearance, V_2_/F apparent peripheral volume compartment, ka first-order absorption rate constant, TLAG lag time for absorption, ηCL is the deviation of clearance for an individual from the population estimate, ηV_1_ is the deviation of the apparent central volume compartment for an individual from the population estimate, ηQ is the deviation of apparent inter-compartmental clearance for an individual from the population estimate, ηV_2_  is the deviation of the apparent peripheral volume compartment for an individual from the population estimate, CV% percentage coefficient variation, σ_add_ additive residual error, σ_prop_ proportional residual error.

**Model fitting and evaluation**

No formal model building or covariate analysis was conducted. The published parameter values provided by Duong et al [1] were used as the initial estimates in the model runs. The following modifications were explored to optimise the model fit;

1. Between subject variability was not supported by the data for inter-compartmental clearance (Q/F) and peripheral volume (V_2_/F) and was fixed to zero.
2. Bioavailability (F) was fixed to 0.55, as per Duong et al 2017 [2]
3. Between occasion variability was tested on F, rather than CL and V and found to be a more stable model so this was retained.
4. To account for samples reported by the lab as below the limit of quantification, a likelihood based method (M3) was used, as described by Ahn et al [3].

As per accepted procedure, weight residual of WRES> 5 was considered an outlier. A total of 17 plasma concentrations met this criteria and were excluded. The final model fit was therefore assessed for n=52 individuals and 395 plasma concentrations.

The fit was assessed to ensure that the model could be considered a reasonable description of the data for the purposes of our analysis, and for the generation of individual estimates of clearance. The fit was based on the biological plausibility of the parameter estimates, the standard errors of parameters, and a prediction-corrected visual predictive check (pcVPC). For the latter, 1000 data sets were simulated under the final model and the 5^th^, 50^th^ and 95^th^ percentiles were plotted against the same percentiles from the original dataset.

The model parameter estimates and the pcVPC are presented in Table S3 and Figures S2. The model provided a suitable fit to the data and the model parameters were estimated with reasonable precision.

S3 Table. Parameter values estimated from the Dunedin and Middlemore data

| Parameter | Values (standard error) |
| --- | --- |
| CL/F (L/h) | 40.2 (3.7) |
| V_1_/F (L) | 71.2 (10.2) |
| Q/F (L/h) | 5.3 (0.53) |
| V_2_/F (L) | 190.8 (24.8) |
| k_a_ (h^-1^) | 0.39 (0.04) |
| TLAG (h) | 0.09 (0.02) |
| F1 | 0.55 (fix) |
| Inter-individual variability | |
| ηCL (CV%) | 45.8 (0.06) |
| ηV_1_ (CV%) | 28.1 (0.06) |
| Covariance of parameters (R) |  |
| ηCL, ηV_1_ | 0.39 |
| Between occasion variability F1 (CV%) | 28.9 (0.02) |
| Residual error | |
| σ_add_ (mg/L) | 0.01 (fix) |
| σ_prop_ (%) | 0.34 (0.01) |

CL/F apparent clearance, V_1_/F apparent central volume compartment, Q/F apparent inter-compartmental clearance, V_2_/F apparent peripheral volume compartment, ka first-order absorption rate constant, TLAG lag time for absorption, ηCL is the deviation of clearance for an individual from the population estimate, ηV_1_ is the deviation of the apparent central volume compartment for an individual from the population estimate, CV% percentage coefficient variation, σ_add_ additive residual error, σ_prop_ proportional residual error.

S2 Figure. Model fit (pcVPCs) for the Dunedin data (top) and Middlemore data (bottom)


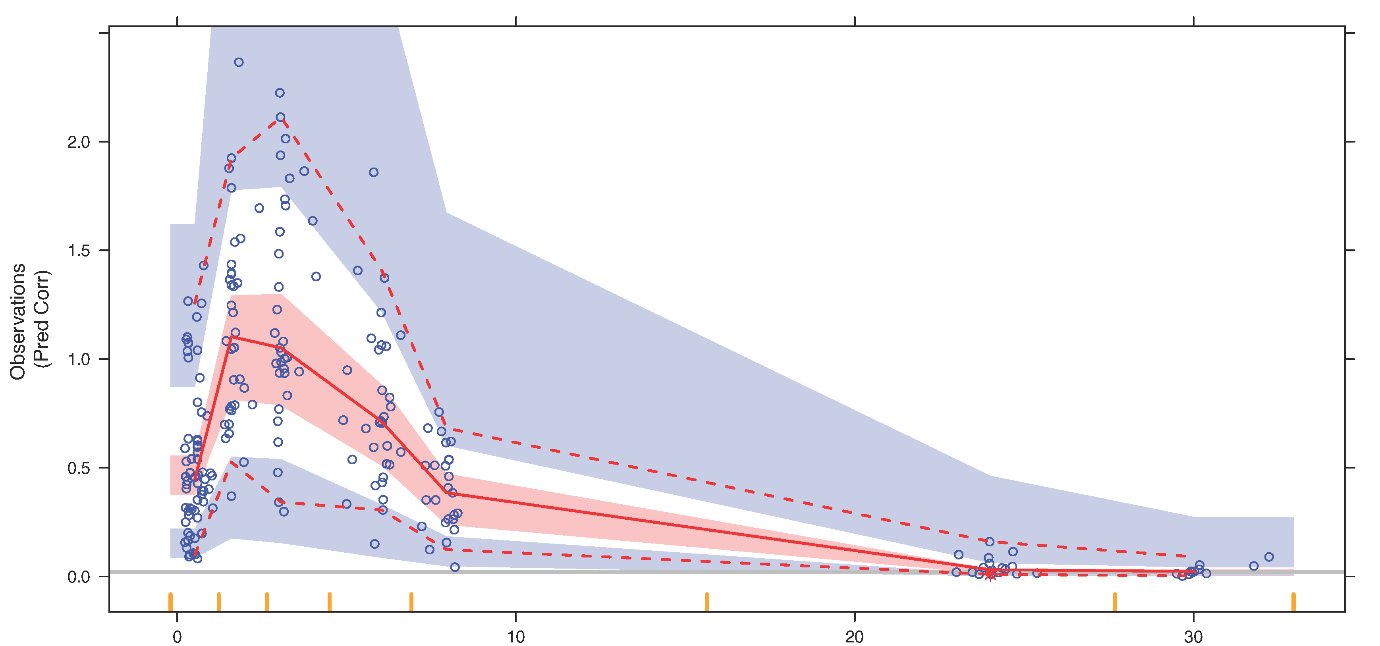


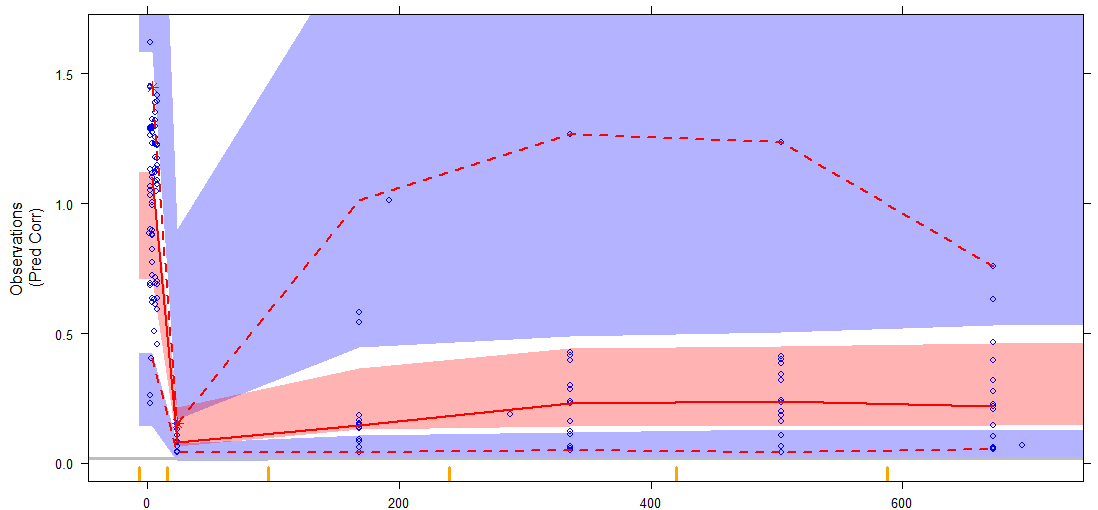


**References**

1. Duong JK, Kumar SS, Kirkpatrick CM, Greenup LC, Arora M, Lee TC et al. Population pharmacokinetics of metformin in healthy subjects and patients with type 2 diabetes mellitus: simulation of doses according to renal function. Clin Pharmacokinet. 2013;52(5):373-84. doi:10.1007/s40262-013-0046-9.

2. Duong JK, Kroonen M, Kumar SS, Heerspink HL, Kirkpatrick CM, Graham GG et al. A dosing algorithm for metformin based on the relationships between exposure and renal clearance of metformin in patients with varying degrees of kidney function. Eur J Clin Pharmacol. 2017;73(8):981-90. doi:10.1007/s00228-017-2251-1.

3. Ahn JE, Karlsson MO, Dunne A, Ludden TM. Likelihood based approaches to handling data below the quantification limit using NONMEM VI. J Pharmacokinet Pharmacodyn. 2008;35(4):401-21. doi:10.1007/s10928-008-9094-4.
