# Supplementary material for: Metformin doses to ensure efficacy and safety in patients with reduced kidney function
Source: PLoS One. 2021 Feb 18;16(2):e0246247. doi: 10.1371/journal.pone.0246247 (PMC7891741; doi:10.1371/journal.pone.0246247)
Supplement: S3 File — (DOCX) [file pone.0246247.s004.docx]

**S3 File. Determination of creatinine clearance and eGFR metrics**

Creatinine clearance was estimated using the Cockcroft and Gault equation (CLcr_CG_) (Equation 7) [1]. In the Cockcroft and Gault equation, age is in years, body weight in kilograms, S_cr_ is serum creatinine in mg/100mL and CLcr_CG_ is creatinine clearance in units of mL/min. Serum creatinine was converted from units of μmol/L to mg/100mL by dividing by 88.42. Creatinine clearance was estimated using the Cockcroft Gault equation in R (version 3.5.3).

${CLcr}_{CG}= \frac{\left( 140-age \right)\left( weight \right)}{72 \cdot S_{cr}} \cdot0.85 \left( if female \right)$ (S3)

Estimated glomerular filtration rate was determined using the 4-variable Modification of Diet in Renal Disease (MDRD) [2] and Chronic Kidney Disease Epidemiology Collaboration (CKD-Epi) [3] creatinine-based estimating equations. In this study, eGFR calculated using the 4-variable MDRD and CKD-Epi equations are referred to as eGFR_MDRD_ and eGFR_CKDEPI_ (Equation S4 and S5, respectively). In equation S5, $\kappa$ is 0.7 for females and 0.9 for males, $\alpha$ is -0.329 for females and -0.411 for males. The calculation of eGFR was performed in R (version 3.5.3).

$e{GFR}_{MDRD}=1.75 \cdot{S_{cr}}^{-1.154} \cdot{age}^{-0.203} \cdot0.742 \left( if female \right)$ (S4)

${eGFR}_{CKDEPI}=141\cdot min\left( {S_{cr}}/{\kappa,1} \right)^{\alpha}\cdot max\left( {S_{cr}}/{\kappa,1} \right)^{-1.209}\cdot{0.993}^{age}\cdot\left[ 1.018 if female \right]\cdot\left[ 1.159 if African American \right]$ (S5)

**References**

1. Cockcroft DW, Gault MH. Prediction of creatinine clearance from serum creatinine. Nephron. 1976;16(1):31-41. doi:10.1159/000180580.

2. Levey AS, Coresh J, Greene T, Stevens LA, Zhang YL, Hendriksen S et al. Using standardized serum creatinine values in the modification of diet in renal disease study equation for estimating glomerular filtration rate. Ann Intern Med. 2006;145(4):247-54. doi:10.7326/0003-4819-145-4-200608150-00004.

3. Levey AS, Stevens LA, Schmid CH, Zhang YL, Castro AF, 3rd, Feldman HI et al. A new equation to estimate glomerular filtration rate. Ann Intern Med. 2009;150(9):604-12. doi:10.7326/0003-4819-150-9-200905050-00006.
