# Supplementary material for: Metformin doses to ensure efficacy and safety in patients with reduced kidney function
Source: PLoS One. 2021 Feb 18;16(2):e0246247. doi: 10.1371/journal.pone.0246247 (PMC7891741; doi:10.1371/journal.pone.0246247)
Supplement: S4 File — S3 Fig. Predicted plasma metformin concentrations at varying levels of renal impairment. (DOCX) [file pone.0246247.s005.docx]

**S4 File. Evaluation of the implemented PK model**

The simulated metformin plasma concentration time profiles in the publication by Duong et al were replicated to ensure that the model was correctly implemented in our hands. Stochastic simulations were performed using the maximum dose for patients with varying levels of renal impairment [1]. The median weight (i.e. 65 kg) for the study population was used for the simulations. Each simulation was replicated 1000 times up to day 25 (presumed steady state) in R (version 3.5.3) using the package RxODE (version 0.9.0-7). The 5th, 50th and 95th percentiles of the simulated concentrations were plotted and compared to the same outputs in the published work (see Figure S3). The replicated predictions from the metformin pharmacokinetic model are visually similar to the published plots produced by Duong et al.

| a | 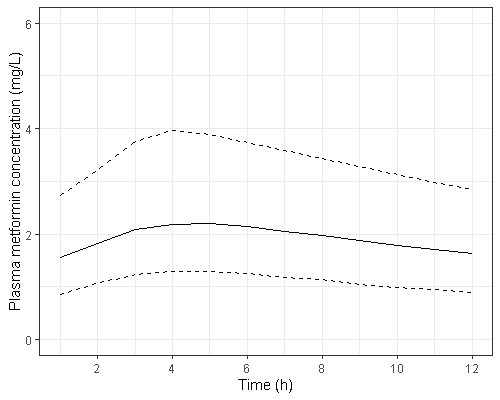 | b | 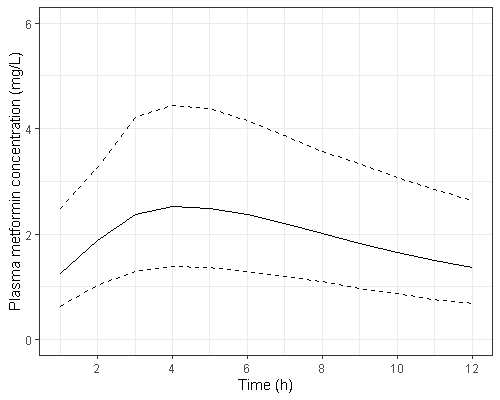 |
| --- | --- | --- | --- |
| c | 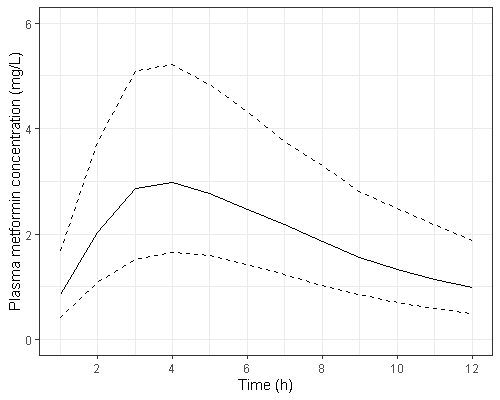 | d | 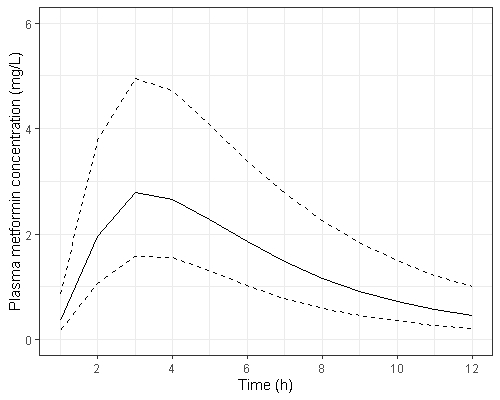 |

S3 Figure. Predicted plasma metformin concentrations at varying levels of renal impairment. a) metformin 500mg, CLcr 15 mL/min, b) metformin 1000 mg CLcr 30 mL/min, c) metformin 2000 mg CLcr 60 mL/min, d) metformin 3000 mg CLcr 120 mL/min. The 5th, 50th and 95th percentiles of the predicted concentrations are shown.

**References**

1. Duong JK, Kumar SS, Kirkpatrick CM, Greenup LC, Arora M, Lee TC et al. Population pharmacokinetics of metformin in healthy subjects and patients with type 2 diabetes mellitus: simulation of doses according to renal function. Clin Pharmacokinet. 2013;52(5):373-84. doi:10.1007/s40262-013-0046-9.
