# Supplementary material for: Metformin doses to ensure efficacy and safety in patients with reduced kidney function
Source: PLoS One. 2021 Feb 18;16(2):e0246247. doi: 10.1371/journal.pone.0246247 (PMC7891741; doi:10.1371/journal.pone.0246247)
Supplement: S5 File — S4 Fig. The clearance for metformin regressed against different measures of kidney function, including (a) CLcrCG, (b) eGFRMDRD, and, (c) eGFRCKDEPI. S4 Table. Summary of linear regression results. (DOCX) [file pone.0246247.s006.docx]

S5 File. Regression analysis details

| **a** | 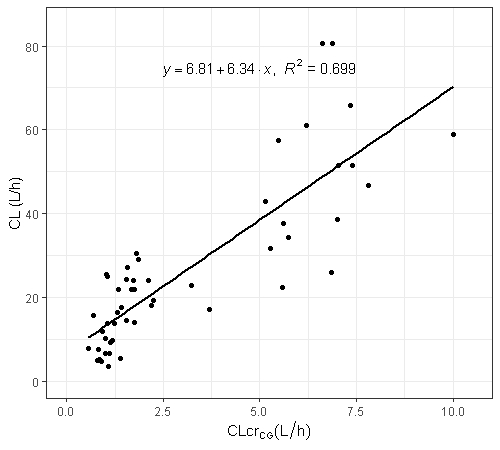 | | **b** | 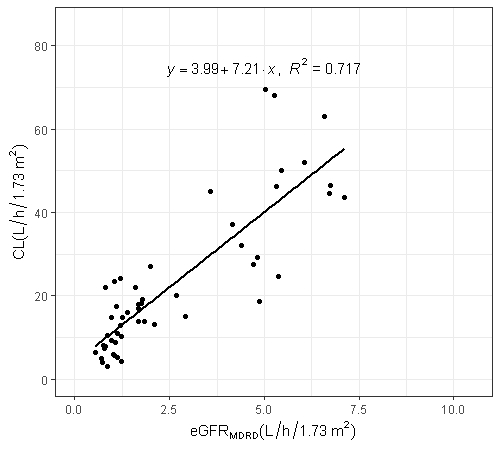 |
| --- | --- | --- | --- | --- |
| **c** | | 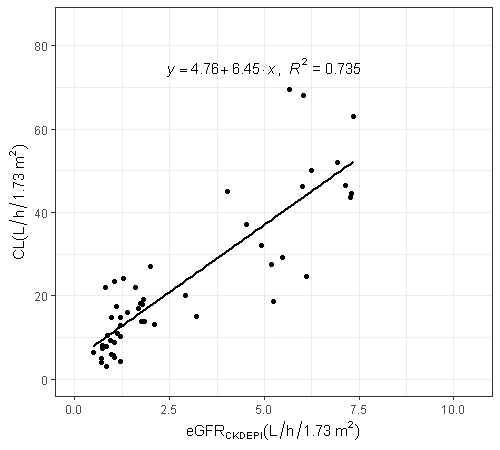 | | |

S4 Figure. The clearance for metformin regressed against different measures of kidney function, including (a) CLcr_CG_, (b) eGFR_MDRD_, and, (c) eGFR_CKDEPI_. Each point represents a study subject.

S4 Table. Summary of linear regression results

| Method | Intercept (95% CI interval) | Slope (95% CI interval) | R-squared value |
| --- | --- | --- | --- |
| CLcr_CG_ | 6.81 (2.16-11.46) | 6.34 (5.18-7.50) | 0.699 |
| eGFR_MDRD_ | 3.99 (-0.17-8.15) | 7.21 (5.94-8.48) | 0.717 |
| eGFR_CKDEPI_ | 4.76 (0.86-8.66) | 6.45 (5.36-7.54) | 0.735 |

CI = confidence interval
